# Mass Azithromycin Provision for Infants in Mali and Survival

**DOI:** 10.1056/NEJMoa2504644

**Published:** 2025-10-16

**Authors:** Fadima Cheick Haidara, Laura Adubra, Mahamadou Abdou, Dagmar Alber, Ulla Ashorn, Yin Bun Cheung, Elaine Cloutman-Green, Mamadou Diallo, Camilla Ducker, Yue-Mei Fan, Gwydion Gruffudd, Lotta Hallamaa, Tiia Haapaniemi, Rikhard Ihamuotila, Jane Juma, Nigel Klein, Juho Luoma, Owen Martell, Akshaya Murugesan, Collins Okello, Oumar Samaké, Cheick Amadou Tidiane Traore, Taru Vehmasto, Kaisa Ylikruuvi, Samba Sow, Per Ashorn

**Affiliations:** 1 Center for Vaccine Development–Mali, Bamako, Mali; 2 Center for Child, Adolescent and Maternal Health Research, Faculty of Medicine and Health Technology, Tampere University and Tampere University Hospital, Tampere, Finland; 3 Ministère de la Santé et du Développement Social du Mali, Bamako, Mali; 4 Great Ormond Street Institute of Child Health, University College London, UK; 5 Great Ormond Street Hospital, London, UK; 6 Tro Da Ltd, Caerphilly, Wales, UK

**Keywords:** Randomized, Infant, Antibiotic, Azithromycin, Mortality, Morbidity, Growth, Infection, Inflammation, Antimicrobial resistance

## Abstract

**Background:**

Mass drug administration (MDA) of azithromycin to 1–59-month-old children reduced mortality in those under five in some sub-Saharan African settings. The largest effects have appeared to be in infants under 12 months, within three months of treatment. Those results led to the present work.

**Methods:**

In this study, we randomly assigned villages in Mali, West Africa, in a 3:4:2 ratio to receive placebo, biannual azithromycin, or quarterly azithromycin. Every three months, 1–11-month-old infants received a single 20 mg/kg dose of placebo (control); azithromycin during January-June and placebo during July-December (biannual); or azithromycin throughout the year (quarterly). The primary outcome was mortality within 90 days of treatment eligibility, expressed as deaths per 1000 person-years at risk (PYR), analyzed as intention-to-treat.

**Results:**

Between December 2020 and December 2022, 1151 villages were enrolled in placebo (n=386), biannual azithromycin (n=511), or quarterly azithromycin (n=254) groups. In all, 149,201 infants received at least one dose of study drug, with 82,600 PYRs of follow-up time and 968 recorded deaths. Mortality rates were 11.9 deaths per 1000 PYR in control, 11.8 (incidence rate ratio, 1.00, 95% confidence interval 0.83 to 1.19) in biannual azithromycin, and 11.3 (0.93, 0.75 to 1.15) in quarterly azithromycin groups, respectively. No effect modification by prespecified child or household factors was observed. Adverse events were rare and similar across groups. Mortality among untreated 12–59-month-olds was similar across groups.

**Conclusion:**

Azithromycin MDA, limited to 1–11-month-old infants, whether delivered biannually or quarterly, did not reduce infant or child mortality in Mali.

Global efforts have halved the under-five mortality rate (U5MR) since 2000, reducing deaths in children to a record low of 4.9 million in 2022. Yet, preventable diseases still claim millions of young lives, particularly during infancy and in high-mortality regions such as the semiarid region of western and north-central Africa called the Sahel (1). With 59 countries unlikely to achieve the 2030 U5MR target of 25 or fewer deaths per 1,000 live births (1,2), the 77^th^ World Health Assembly called for urgent action to scale up evidence-based, cost-effective interventions that would accelerate progress in reducing preventable child mortality (3).

One promising intervention is mass drug administration (MDA) of azithromycin, a broad-spectrum antibiotic initially used for trachoma control (4,5). Azithromycin MDA has shown potential in reducing the prevalence of infections such as malaria, diarrhea, and pneumonia — leading causes of child mortality in high-burden settings (6–12). Mortality reductions were also observed in trachoma trials (13–15). The Macrolides Oraux pour Réduire les Décès avec un Oeil sur la Résistance (MORDOR) trial, conducted in Niger, Malawi, and Tanzania, specifically examined the impact of biannual azithromycin MDA on mortality in children aged 1–59 months (4). The results showed a statistically significant 13.5% reduction in all-cause mortality, 6.6 fewer deaths per 1,000 person-years—with the largest reductions among infants aged 1–5 months (24.9% lower mortality) and in Niger (18.1%). A secondary analysis suggested that much of the protective effect was in the first three months post-treatment (16), raising important considerations for optimal dosing frequency.

Following these findings, the World Health Organization (WHO) issued conditional guidelines in 2020, recommending consideration of azithromycin MDA for 1–11-month-old infants in high-mortality settings (17). This age restriction was designed to maximize benefits while minimizing the risks of antimicrobial resistance (AMR) (17–19).

In the present trial, the **L**arge-scale **A**ssessment of the **K**ey health-promoting **A**ctivities of two **N**ew mass drug administration regimens with **A**zithromycin (LAKANA) trial, we evaluated the effects of azithromycin MDA in Mali, West Africa, targeting 1-to-11-month-old infants and provided either twice or four times a year. This article reports the trial results on infant and child mortality, as well as intervention safety.

## METHODS

### Trial Design and Oversight

Our trial was a cluster-randomized, placebo-controlled, double-blind, parallel-group, three-arm clinical trial, with an adaptive design that compared the effects of azithromycin (Zithromax, Pfizer) to placebo, administered to 1–11-month-old infants (29–364 days), on infant and child mortality in Mali. We assessed all-cause mortality over two years in villages randomized in a 3:4:2 ratio to control, biannual azithromycin, and quarterly azithromycin. Every three months, infants in the control arm received placebo, while those in the quarterly azithromycin group received azithromycin. In the biannual azithromycin group, infants received azithromycin twice at three-month intervals between January and June —avoiding overlap with the national seasonal malaria chemoprevention (SMC) program, which runs from July to December—and placebo twice at three-month intervals between July and December.

Details of the Methods can be found in the protocol, available with the full text of this article at NEJM.org, and were previously reported(20).

The principal investigators (see protocol at NEJM.org) designed the trial, ensured protocol fidelity, decided to publish, and vouch for the accuracy of the data and analysis. Per Ashorn wrote the first manuscript draft; Juho Luoma and Lotta Hallamaa conducted data analysis. All authors revised the manuscript and approved the submission.

Independent expert committees advised the investigators on design and implementation, while a data and safety monitoring board (DSMB) monitored trial progress and safety.

Ethics approval was obtained from the Mali Institutional Review Board, the Comité d’Éthique de l’Université des Sciences, des Techniques et des Technologies de Bamako. The trial also received a favorable opinion from the Ethics Committee of the Pirkanmaa Hospital District for Tampere University researchers to participate in the project.

Permissions were granted by village leaders, and verbal consent—recorded as ‘yes’ or ‘no’ and confirmed with a digital signature on tablets—was obtained from household heads or authorized proxies before activities began. Verbal consent was obtained from at least one caregiver for treatment of infants.

### Trial Setting, Participants, and Eligibility Criteria

The present trial covered villages located in the Kayes, Kita, and Koulikoro regions considered non-urban, accessible, and safe by the local health authorities and research team. Eligibility for study drug administration included age 1–11 months (29–364 days), residence in a trial village, weight of at least 3.0 kg, and parental consent. Infants with known macrolide allergies, or severe illness requiring referral were excluded.

### Randomization and Masking

The randomization unit (cluster) was the village, with all infants in a given village receiving the same treatment. Randomization was stratified by cluster size —below or above 100 infants —based on national population estimates. Eighteen letters were randomly assigned to azithromycin or placebo and used in combinations to allocate villages to control, biannual, or quarterly treatment. Villages were randomly allocated into the trial arms at public events where village representatives blindly pulled two-letter coded lottery tickets (Details in Supplementary Appendix 1 and at lakana.org).

Unmasked personnel included staff from Research Triangle Institute International, Pfizer, an external statistician, and the DSMB chair. Study arm allocation was masked to participants, site staff, investigators, and the statistician who led the analysis. Azithromycin and placebo were similar in appearance, smell, and packaging to maintain blinding.

### Census and Follow-Up

A house-to-house census was performed every three months in each village over two years, to enumerate the population and provide study drug. A flexible ± four-week window was allowed around each three-month interval.

At the first household visit, the trial team conducted a baseline assessment, collecting Global Positioning System coordinates, socio-economic and Water, Sanitation, and Hygiene (WASH) data, and demographic information. At subsequent visits, vital status of all household members was updated, and new members and households were added. Vital status was categorized as alive, deceased, moved, or unknown. Data collectors also recorded infants’ exposure to SMC and immunizations.

All data were collected using a mobile application (CommCare by Dimagi, Cambridge, MA, USA).

### Interventions

At each MDA visit, infants were weighed on an electronic hanging scale (ADE Model M111600–01, Hamburg, Germany), and the study mobile application calculated the dose in milliliters to be administered. Data collectors used syringes to administer oral azithromycin and placebo suspension, under direct observation and at a single dose of 20 mg per kilogram of body weight, in line with current WHO guidelines(17). Each infant could receive a total of 1–4 doses of study drug, depending on their age at enrollment. If vomiting occurred within 15 minutes of ingestion, a new dose of the same size was given.

### Outcomes

The prespecified primary outcome was all-cause mortality rate: deaths per 1000 person-years at risk (PYR) among 1–11-month-old (29–364 days) infants. The unit of primary outcome measurement was a three-month time interval, between successive study visits. The dates of the consecutive visits were used to calculate PYR. Any child could contribute 1–4-time intervals to the primary outcome analysis.

The secondary outcomes focused on indirect effects. We assessed all-cause mortality among children aged 12–59 months at the latest azithromycin MDA in their village. We also assessed effect modification by the child’s age at the time of MDA, along with sex and weight-for-age z-score (WAZ); seasonality; SMC exposure; order of MDA; district of residence; distance from the nearest health facility in km; household asset index; WASH index; and national outreach strategy (Supplementary Appendix Table S1).

### Adverse Events

Serious adverse events (SAEs) included any adverse events resulting in death, life-threatening condition, hospitalization or prolongation of existing hospitalization, significant disability or incapacity, or any condition deemed medically important by a study physician. Adverse events (AEs) included any new illness or symptom within 14 days post-MDA.

SAEs were monitored through passive surveillance at all study sites. Caregivers were advised to report major symptoms occurring within 14 days of treatment, and health center agents to report deaths, or hospitalizations. Deaths occurring more than 14 days post-MDA or identified at subsequent visits were reported as primary outcomes. AEs were monitored through active surveillance via caregiver interviews 14 days post-MDA for 4–11-month-olds in a subset sample of 59 villages selected for other study objectives.

### Statistical Analysis

All enrolled households and infants treated at least once were included in the study database. We analyzed mortality outcomes using the intention-to-treat (ITT) principle(21). Power-by-simulation was used to determine the required sample size for the primary outcome analyses and to confirm that interim analysis-related procedures did not compromise type I error rates(22). A sample size of 1151 clusters with on average 31 analyzable infants per cluster per time interval provided approximately 89% power for testing the hypothesis that biannual azithromycin MDA reduced mortality, >99% power for testing the hypothesis that quarterly azithromycin MDA reduced mortality, and 80% power for testing the hypothesis that quarterly azithromycin MDA reduced mortality more than biannual azithromycin MDA.

A prespecified interim analysis was conducted by an independent statistician and reviewed by the DSMB when approximately 60% of the estimated total person-years had accrued(20,23).

The trial hypotheses were that, first, infant mortality would be lower in villages receiving biannual azithromycin MDA than in control villages, and second, would be lower in villages receiving quarterly azithromycin MDA than in those receiving biannual azithromycin. We conducted hypothesis testing for the primary outcome as one-sided. We estimated incidence rate ratios (IRRs) and 95% confidence intervals (CIs) to compare treatment regimens. We used mixed-effects Poisson modelling to estimate intervention effects between treatment groups, with random intercepts for clusters, using log link function with person-years as an offset variable. Models were adjusted for the randomization stratification factors (cluster size) as a fixed effect. As per the close-testing method for controlling multiple group comparisons, the global null hypothesis of mortality in all three groups being the same was tested at a 5% significance level. A pairwise null hypothesis was rejected at one-sided p < 0.025 only if the global null hypothesis was rejected. Analyses of secondary outcomes were conducted as two-sided tests, with two-sided p-values and 95% CIs.

Effect modification was assessed using mixed-effects Poisson models with an interaction term between the intervention and each modifier. Stratified comparisons were tested only if the interaction was significant (p < 0.1).

All analyses were conducted using Stata v18.0 (Stata Corp LLC, College Station, TX, USA) and R v4.3.2 (R Foundation for Statistical Computing, Vienna, Austria).

## RESULTS

### Enrollment and Baseline Characteristics

**Figure 1.**
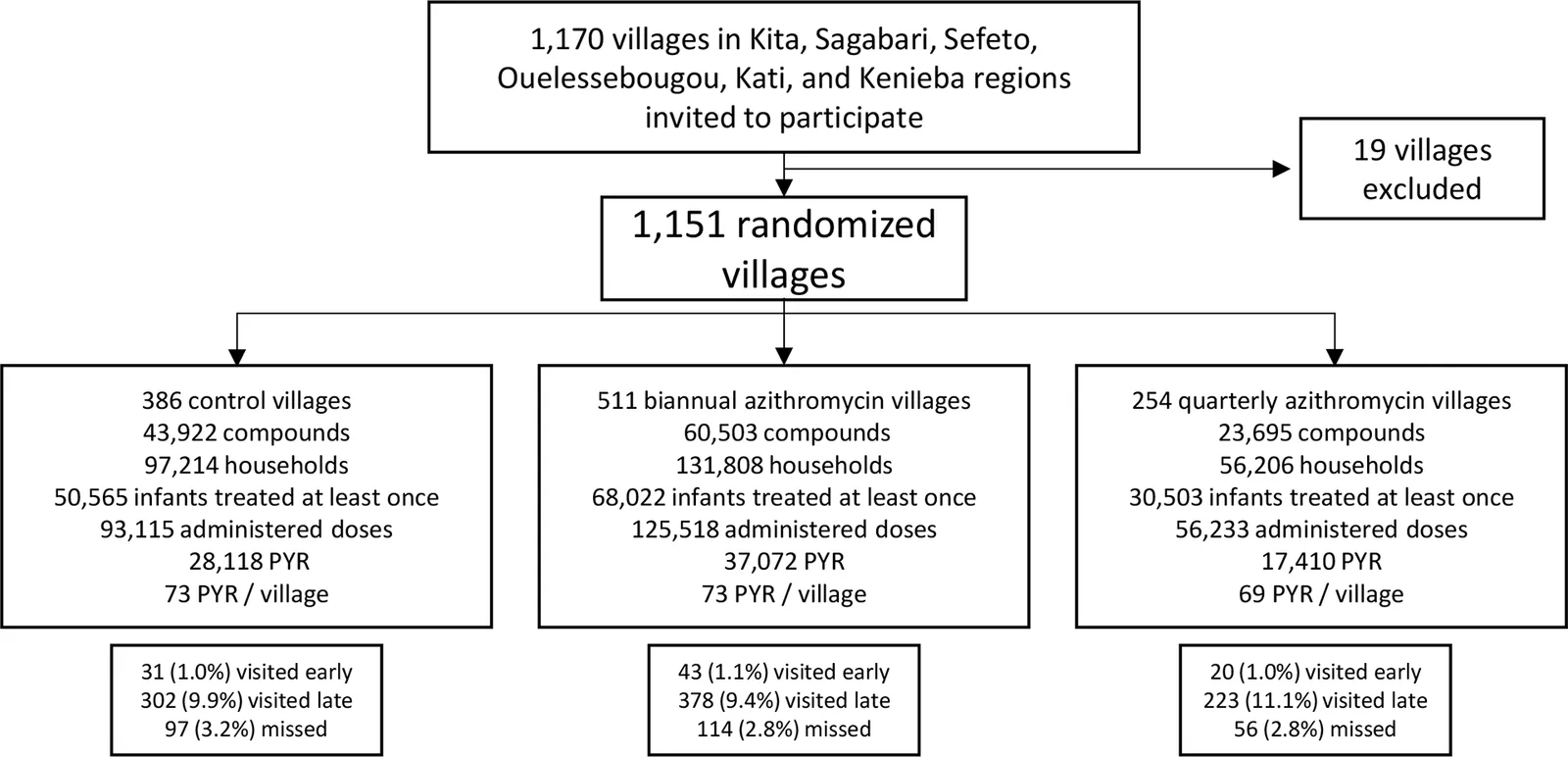
Trial flow chart The term “compounds” refers to local residential groupings that consist of multiple households in the study setting. Abbreviation: PYR person-years at risk.

Between November 2020 and December 2022, 1,170 villages in 11 administrative districts were screened; 19 were ineligible or declined participation. The remaining 1151 villages were randomized to control (386), biannual azithromycin (511), or quarterly azithromycin (254) (Fig. 1, Figs.S1 and S2).

At the first MDA, the groups were similar in village size, number of eligible infants per village, and infant age, sex, and weight-for age distributions (Table 1).

### Delivery of the Study Drug

Of the 9,085 planned MDA visits, 86.2% were completed on time, 1.0% early, 9.9% late, and 2.9% were missed. Completion proportions were similar between trial arms and varied slightly by MDA visits (Fig. S1). The last MDA round in 128 villages was not completed due to study drug expiry; in 10 additional villages, follow-up was truncated for other reasons.

Over the trial period, 285,227 households were registered; 27 households declined participation. A total of 149,201 infants received at least one dose of study drug, with 274,896 recorded treatments and 82,600 PYR of follow-up time (Figure 1). Vital status was unknown for 4,080 (2.7%) treated infants due to migration or other loss to follow-up: 2.8% in the control group, 2.7% in the biannual azithromycin group, and 2.6% in the quarterly azithromycin group.

The study drug could not be administered as planned in 10,094 (3.7%) of treatment visits, mostly due to temporary absence, weight below 3 kg, illness, allergy concerns, or caregiver refusal. In 273 (0.1%) treatments, the infant erroneously received study preparation with an incorrect letter code. In 41 of these events, the error affected the actual treatment received. In 1,930 (0.7%) treatments, infants received a second dose due to vomiting within 15 minutes of the first. All the proportions were similar across groups (Table S2).

### Primary Outcomes

During the study, 968 deaths were recorded among infants aged 1–11 months at the time of MDA (Table 2, Tables S3 and S4). The overall mortality was 11.7 deaths per 1,000 PYR; 11.9 in the control, 11.8 in the biannual azithromycin and 11.3 in the quarterly azithromycin group (p = 0.76). Compared to the control, the IRR (95% CI) for mortality among infants aged 1–11 months at the time of MDA was 1.00 (0.83 to 1.19) in the biannual azithromycin group and 0.93 (0.75 to 1.15) in the quarterly azithromycin group. Compared to the biannual azithromycin group, the respective IRR in the quarterly azithromycin group was 0.93 (0.76 to 1.15). Absolute between-group mortality differences were minimal.

### Secondary Outcomes

The mortality ratio between the control and either azithromycin group among infants aged 1–11 months at the time of MDA was not modified by infant age, sex, WAZ, recent SMC dosing, household asset or WASH index (Fig, 2 and Fig, S3). Similarly, there was no effect modification by seasonality, MDA order, distance to the nearest health facility or national outreach strategy. Some variation by district was observed, with a few statistically significant but inconsistent differences between groups (Figs. S4 and S5).

There were 967 deaths among untreated children aged 12–59 months at the time of MDA. Adjusted for follow-up time, the overall mortality was 10.6 deaths per 1000 PYR among 12–23-month-olds, 3.4 among 24–35-month-olds, 2.2 among 36–47-month-olds, and 1.5 among 48–59-month-olds. Compared to the control, the IRR (95% CI) for mortality among children aged 12–59 months at the time of MDA was 1.03 (0.85 to 1.24) in the biannual azithromycin group and 0.97 (0.77 to 1.22) in the quarterly azithromycin group (Table S5).

### Adverse Events

No suspected SAEs were reported. Among 1,408 infants with active AE monitoring, caregivers reported 22 episodes of diarrhea, loose stools or vomiting, 12 episodes of crying more than usual, and 31 other episodes. Adverse events occurred in 3.2% of infants in the control, 1.0% in the biannual azithromycin group, and 1.9% in the quarterly azithromycin group.

## DISCUSSION

We evaluated the impact of biannual and quarterly MDA with azithromycin on infant and child mortality, when given to 1–11-month-old infants in a high-mortality, holoendemic malaria setting with a national seasonal malaria chemoprevention program. In a Malian sample of 1151 villages, 82,600 person-years of observation and 968 recorded deaths, mortality was not lower in the biannual azithromycin group than the control. Mortality was 7% lower in the quarterly azithromycin group, but the confidence interval around the point-estimate was wide and included the null value. Although the trial was large, mortality was lower than anticipated, and the evidence does not support the hypothesized level of mortality reduction (23). Some subgroup data suggested a potential effect, but random error cannot be excluded. Mortality among untreated 12–59-month-olds was similar across groups.

The results of our trial align with the AVENIR trial in Niger, which also found no significant mortality reduction when biannual azithromycin MDA was targeted at 1–11-month-old infants alone(24). Furthermore, the CHAT and NAITRE trials in Burkina Faso, which provided azithromycin to healthy infants at routine well-child visits in their first three months of life, showed no mortality benefit (25,26). In contrast, both MORDOR and AVENIR trials in Niger observed a 14–18% reduction in mortality when biannual azithromycin MDA was delivered to all 1–59-month-old children (4,24). In the CHAT trial, targeting the same wider age group biannually in Burkina Faso, mortality was 18% lower in azithromycin than placebo clusters, although the difference was not statistically significant (p=0.07) (26).

Taken together, the current evidence suggests that azithromycin MDA reduces mortality among West African children if distributed to all 1–59-month-olds, but not when limited to infants. Whereas the mechanism remains uncertain, containment of infectious diseases is one likely pathway (27–29). Azithromycin has both antibacterial and anti-malarial activity (6–11,30,31), and malaria accounts for a higher proportion of deaths in older children than in infants (32). Older children also harbor more often potentially pathogenic Gram-negative bacteria in the gut than breast-fed infants (33,34), which may contribute to sepsis and be reduced by azithromycin’s antibacterial and anti-inflammatory properties (35–37). Individual-level elimination of potential pathogens would also reduce the community burden of malaria and other infections, explaining the observed herd-protection among infants when including older children in the azithromycin MDA (24).

Lower-than-expected mortality limited power to detect modest differences between groups and may reduce generalizability to higher-mortality settings. In the control, mortality was 12 deaths per 1,000 PYR, corresponding to an IMR of approximately 36 per 1,000—markedly below the initial assumption of 70 per 1,000 based on 2018 data (38), even after adjusting to 60 per 1,000 and increasing the sample size (20,23). A separate trial in Mali documented a major under-five mortality decrease in a control group receiving no intervention, from 148 to 55 deaths per 1,000 between 2017 and 2020 (39). These results suggest a possible Hawthorne-effect on mortality (40) but may also reflect a national reduction in under-five mortality (41), which could partially explain the lack of effect observed in our trial (42). (Representativeness is further addressed in Table S6).

WHO currently recommends targeting 1–11-month-old infants if azithromycin MDA is considered for child survival (17). While this focused approach will certainly limit the risk of AMR, the latest evidence suggests that a mortality benefit may require targeting a wider age group. However, the optimal target group is still unknown, due to limited data and lack of clarity on the intervention’s mechanism of action (17). In any case, monitoring AMR prevalence will be important if azithromycin MDA is scaled up (43), as currently happening in Nigeria, Niger and Mali (44–46). Tracking mortality trends will also be essential, as the intervention’s efficacy may wane with declining baseline mortality (42).

In conclusion, azithromycin MDA, given to 1–11-month-old infants either biannually or quarterly, did not reduce infant or child mortality in Mali.

## Supplementary Material

supplement

## Figures and Tables

**Figure 2.**
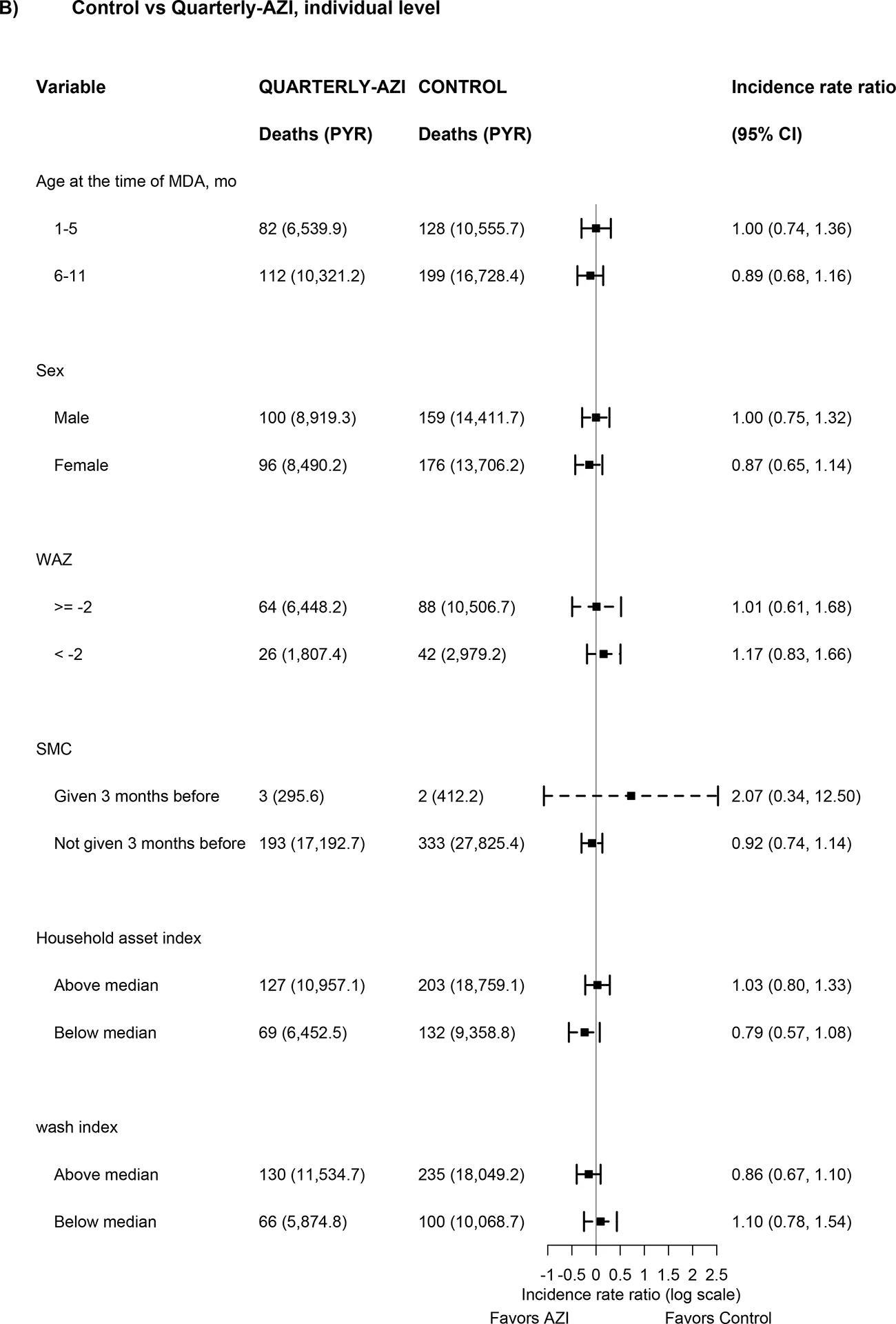
Mortality difference between the control and quarterly azithromycin groups, among predefined sub-groups of infants, who were 1 – 11-months old at the time of mass-drug administration, according to individual and household characteristics. Abbreviations: AZI azithromycin; CI confidence interval; MDA mass drug administration; PYR person-years at risk; SMC Seasonal Malaria Chemoprevention; WASH Water, Sanitation, and Hygiene; WAZ Weight-for-Age Z-score

**Table 1.** Characteristics of the study communities and 1 – 11-month-old infants at the first mass drug administration, by trial arm

	Control	Biannual azithromycin	Quarterly azithromycin
Number of villages	386	511	254
Number (%) of large villages	70 (18%)	88 (17%)	44 (17%)
Median number of 1–11 mo. infants / village	17	18	19
Total number of 1–11 mo. infants	13,187	18,040	8,201
Proportion of girls	49.3%	49.0%	49.4%
Mean (SD) WAZ	−0.89 (1.35)	−0.89 (1.38)	−0.92 (1.36)
Mean (SD) age, months	6.0 (3.0)	6.0 (3.1)	6.1 (3.1)
Proportion in different age groups			
1–2 months	18.7%	19.0%	18.4%
3–5 months	29.8%	29.6%	29.3%
6–8 months	29.8%	28.8%	29.5%
9–11 months	21.7%	22.6%	22.8%

Abbreviations: SD = Standard deviation, WAZ = weight for age Z-score

**Table 2.** All-cause mortality among participants who were 1 – 11-months old at the time of mass-drug administration.

	Control	Biannual azithromycin	Quarterly azithromycin	
Number of deaths	335	437	196	
Number of PYR	28,117.9	37,072.0	17,409.5	
Number of deaths/1000 PYR	11.9	11.8	11.3	
				Quarterly vs Biannual
Incidence rate ratio^1^	REF	1.00	0.93	0.93
(95% CI)		(0.83 to 1.19)	(0.75 to 1.15)	(0.76 to 1.15)
p-value		0.48	0.25	0.25
Incidence rate difference	REF	−0.05	−0.91	−0.85
(95% CI)		(−2.31 to 2.21)	(−3.55 to 1.73)	(−3.34 to 1.64)
p-value		0.96	0.50	0.50

1 Adjusted for stratified randomization factor (village size category)

Abbreviations: CI confidence interval; REF reference; PYR person-years at risk.

## References

[1] United Nations Inter-Agency Group for Child Mortality Estimation (UN IGME). Levels & Trends in Child Mortality: Report 2023, Estimates developed by the United Nations Inter-agency Group for Child Mortality Estimation. New York: United Nations Children’s Fund, New York, 2024.; 2024.

[2] UN General Assembly. Transforming our world: the 2030 agenda for sustainable development [Internet]. 2015 [cited 2024 Dec 16]. Available from: https://www.refworld.org/docid/57b6e3e44.html

[3] World Health Organization (WHO). Accelerating progress towards reducing maternal, newborn and child mortality in order to achieve Sustainable Development Goal targets 3.1 and 3.2 [Internet]. Geneva: Seventy-seventh World Health Assembly, World Health Organization; 2024 [cited 2024 Dec 16]. Available from: https://apps.who.int/gb/ebwha/pdf_files/WHA77/A77_ACONF5-en.pdf

[4] Keenan JD, Bailey RL, West SK, Arzika AM, Hart J, Weaver J, Azithromycin to Reduce Childhood Mortality in Sub-Saharan Africa. N Engl J Med. 2018 Apr 26;378(17):1583–92. 29694816 10.1056/NEJMoa1715474 PMC5849140

[5] Emerson PM, Hooper PJ, Sarah V. Progress and projections in the program to eliminate trachoma. PLoS Negl Trop Dis. 2017 avr;11(4):e0005402. 28426814 10.1371/journal.pntd.0005402 PMC5398479

[6] Whitty CJ, Glasgow KW, Sadiq ST, Mabey DC, Bailey R. Impact of community-based mass treatment for trachoma with oral azithromycin on general morbidity in Gambian children. Pediatr Infect Dis J. 1999 Nov;18(11):955–8. 10571428 10.1097/00006454-199911000-00003

[7] Fry AM, Jha HC, Lietman TM, Chaudhary JSP, Bhatta RC, Elliott J, Adverse and beneficial secondary effects of mass treatment with azithromycin to eliminate blindness due to trachoma in Nepal. Clin Infect Dis Off Publ Infect Dis Soc Am. 2002 Aug 15;35(4):395–402. 10.1086/341414 12145722

[8] Coles CL, Seidman JC, Levens J, Mkocha H, Munoz B, West S. Association of mass treatment with azithromycin in trachoma-endemic communities with short-term reduced risk of diarrhea in young children. Am J Trop Med Hyg. 2011 Oct;85(4):691–6. 21976574 10.4269/ajtmh.2011.11-0046 PMC3183779

[9] Coles CL, Levens J, Seidman JC, Mkocha H, Munoz B, West S. Mass distribution of azithromycin for trachoma control is associated with short-term reduction in risk of acute lower respiratory infection in young children. Pediatr Infect Dis J. 2012 Apr 1;31(4):341–6. 22173140 10.1097/INF.0b013e31824155c9

[10] Gaynor BD, Amza A, Kadri B, Nassirou B, Lawan O, Maman L, Impact of Mass Azithromycin Distribution on Malaria Parasitemia during the Low-Transmission Season in Niger: A Cluster-Randomized Trial. Am J Trop Med Hyg. 2014 May 7;90(5):846–51. 24615132 10.4269/ajtmh.13-0379 PMC4015576

[11] Schachterle SE, Mtove G, Levens JP, Clemens E, Shi L, Raj A, Short-Term Malaria Reduction by Single-Dose Azithromycin during Mass Drug Administration for Trachoma, Tanzania. Emerg Infect Dis. 2014 Jun;20(6):941–9. 24865642 10.3201/eid2006.131302 PMC4036785

[12] Li J, Xiong T, Yue Y, Choonara I, Qazi S, Tang J, Secondary Effects from Mass Azithromycin Administration: A Systematic Review and Meta-analysis. Am J Trop Med Hyg. 2022 Oct 12;107(4):904–11. 35970284 10.4269/ajtmh.22-0134 PMC9651525

[13] Porco TC, Gebre T, Ayele B, House J, Keenan J, Zhou Z, Effect of Mass Distribution of Azithromycin for Trachoma Control on Overall Mortality in Ethiopian Children: A Randomized Trial. JAMA. 2009 Sep 2;302(9):962–8. 19724043 10.1001/jama.2009.1266

[14] Keenan JD, Ayele B, Gebre T, Zerihun M, Zhou Z, House JI, Childhood mortality in a cohort treated with mass azithromycin for trachoma. Clin Infect Dis Off Publ Infect Dis Soc Am. 2011 Apr 1;52(7):883–8. 10.1093/cid/cir069 PMC3106233 21427395

[15] O’Brien KS, Cotter SY, Amza A, Kadri B, Nassirou B, Stoller NE, Childhood Mortality After Mass Distribution of Azithromycin: A Secondary Analysis of the PRET Cluster-randomized Trial in Niger. Pediatr Infect Dis J. 2018;37(11):1082–6. 29561511 10.1097/INF.0000000000001992 PMC6138579

[16] Porco TC, Hart J, Arzika AM, Weaver J, Kalua K, Mrango Z, Mass Oral Azithromycin for Childhood Mortality: Timing of Death After Distribution in the MORDOR Trial. Clin Infect Dis Off Publ Infect Dis Soc Am. 2019 May 30;68(12):2114–6. 10.1093/cid/ciy973 PMC6541729 30561577

[17] World Health Organization. WHO guideline on mass drug administration of azithromycin to children under five years of age to promote child survival [Internet]. Geneva: World Health Organization; 2020 [cited 2022 Mar 24]. 44 p. Available from: https://apps.who.int/iris/handle/10665/333942 32924384

[18] Doan T, Arzika AM, Hinterwirth A, Maliki R, Zhong L, Cummings S, Macrolide Resistance in MORDOR I - A Cluster-Randomized Trial in Niger. N Engl J Med. 2019 Jun 6;380(23):2271–3. 31167060 10.1056/NEJMc1901535 PMC6518950

[19] O’Brien KS, Emerson P, Hooper PJ, Reingold AL, Dennis EG, Keenan JD, Antimicrobial resistance following mass azithromycin distribution for trachoma: a systematic review. Lancet Infect Dis. 2019 Jan;19(1):e14–25. 30292480 10.1016/S1473-3099(18)30444-4

[20] Adubra L, Alber D, Ashorn P, Ashorn U, Cheung YB, Cloutman-Green E, Testing the effects of mass drug administration of azithromycin on mortality and other outcomes among 1–11-month-old infants in Mali (LAKANA): study protocol for a cluster-randomized, placebo-controlled, double-blinded, parallel-group, three-arm clinical trial. Trials. 2023 Jan 3;24(1):5. 36597115 10.1186/s13063-022-06966-7 PMC9809521

[21] McCoy CE. Understanding the Intention-to-treat Principle in Randomized Controlled Trials. West J Emerg Med Integrating Emerg Care Popul Health [Internet]. 2017 [cited 2025 Jan 30];18(6). Available from: https://escholarship.org/uc/item/83j2g4hq 10.5811/westjem.2017.8.35985 PMC5654877 29085540

[22] Feiveson AH. Power by Simulation. Stata J Promot Commun Stat Stata. 2002 Jun;2(2):107–24.

[23] Luoma J, Adubra L, Alber D, Ashorn P, Ashorn U, Cloutman-Green E, Statistical analysis plan for the LAKANA trial: a cluster-randomized, placebo-controlled, double-blinded, parallel group, three-arm clinical trial testing the effects of mass drug administration of azithromycin on mortality and other outcomes among 1–11-month-old infants in Mali. Trials. 2023 Nov 15;24(1):733. 37968741 10.1186/s13063-023-07771-6 PMC10648361

[24] O’Brien KS, Arzika AM, Amza A, Maliki R, Aichatou B, Bello IM, Azithromycin to Reduce Mortality — An Adaptive Cluster-Randomized Trial. N Engl J Med. 2024 Aug 22;391(8):699–709. 39167806 10.1056/NEJMoa2312093

[25] Sié A, Ouattara M, Bountogo M, Boudo V, Ouedraogo T, Compaoré G, Azithromycin during Routine Well-Infant Visits to Prevent Death. N Engl J Med. 2024 Jan 17;390(3):221–9. 38231623 10.1056/NEJMoa2309495

[26] Oldenburg CE, Ouattara M, Bountogo M, Boudo V, Ouedraogo T, Compaoré G, Mass Azithromycin Distribution to Prevent Child Mortality in Burkina Faso: The CHAT Randomized Clinical Trial. JAMA. 2024 Feb 13;331(6):482–90. 38349371 10.1001/jama.2023.27393 PMC10865159

[27] Keenan JD, Arzika AM, Maliki R, Elh Adamou S, Ibrahim F, Kiemago M, Cause-specific mortality of children younger than 5 years in communities receiving biannual mass azithromycin treatment in Niger: verbal autopsy results from a cluster-randomised controlled trial. Lancet Glob Health. 2020 Feb;8(2):e288–95. 31981558 10.1016/S2214-109X(19)30540-6 PMC7025321

[28] Bloch EM, Mrango Z, Weaver J, Munoz B, Lietman TM, West SK. Causes of death after biannual azithromycin treatment: A community-level randomized clinical trial. PLOS ONE. 2021 Sep 24;16(9):e0250197. 34559801 10.1371/journal.pone.0250197 PMC8462712

[29] Hart JD, Kalua K, Keenan JD, Lietman TM, Bailey RL. Effect of Mass Treatment with Azithromycin on Causes of Death in Children in Malawi: Secondary Analysis from the MORDOR Trial. Am J Trop Med Hyg. 2020 Sep;103(3):1319–28. 32342837 10.4269/ajtmh.19-0613 PMC7470551

[30] Rosenthal PJ. Azithromycin for Malaria? Am J Trop Med Hyg. 2016 Jul 6;95(1):2–4. 27215296 10.4269/ajtmh.16-0332 PMC4944689

[31] Chandramohan D, Dicko A, Zongo I, Sagara I, Cairns M, Kuepfer I, Effect of Adding Azithromycin to Seasonal Malaria Chemoprevention. N Engl J Med. 2019 Jun 6;380(23):2197–206. 30699301 10.1056/NEJMoa1811400

[32] Ogbuanu IU, Otieno K, Varo R, Sow SO, Ojulong J, Duduyemi B, Burden of child mortality from malaria in high endemic areas: Results from the CHAMPS network using minimally invasive tissue sampling. J Infect [Internet]. 2024 Mar 1 [cited 2025 Feb 24];88(3). Available from: https://www.journalofinfection.com/article/S0163-4453(24)00025-2/fulltext 10.1016/j.jinf.2024.01.006 PMC13019364 38290664

[33] Robertson RC, Manges AR, Finlay BB, Prendergast AJ. The Human Microbiome and Child Growth – First 1000 Days and Beyond. Trends Microbiol. 2019 Feb 1;27(2):131–47. 30529020 10.1016/j.tim.2018.09.008

[34] Stewart CJ, Ajami NJ, O’Brien JL, Hutchinson DS, Smith DP, Wong MC, Temporal development of the gut microbiome in early childhood from the TEDDY study. Nature. 2018 Oct;562(7728):583–8. 30356187 10.1038/s41586-018-0617-x PMC6415775

[35] Kanoh S, Rubin BK. Mechanisms of Action and Clinical Application of Macrolides as Immunomodulatory Medications. Clin Microbiol Rev. 2010 Jul;23(3):590–615. 20610825 10.1128/CMR.00078-09 PMC2901655

[36] Pavlinac PB, Platts-Mills JA, Liu J, Atlas HE, Gratz J, Operario D, Azithromycin for Bacterial Watery Diarrhea: A Reanalysis of the Antibiotics for Children with Severe Diarrhea (ABCD) Trial Incorporating Molecular Diagnostics. J Infect Dis. 2024 Apr 12;229(4):988–98. 37405406 10.1093/infdis/jiad252 PMC11011181

[37] Doan T, Hinterwirth A, Worden L, Arzika AM, Maliki R, Abdou A, Gut microbiome alteration in MORDOR I: a community-randomized trial of mass azithromycin distribution. Nat Med. 2019 Sep;25(9):1370–6. 31406349 10.1038/s41591-019-0533-0

[38] Institut National de la Statistique (INSTAT) and ICF. 2018 Mali Demographic and Health Survey Key Findings. Rockville, Maryland, USA: INSTAT and ICF; 2019.

[39] Liu J, Treleaven E, Whidden C, Doumbia S, Kone N, Beydi Cisse A, Home visits versus fixed-site care by community health workers and child survival: a cluster-randomized trial, Mali. Bull World Health Organ. 2024 Sep 1;102(9):639–49. 39219760 10.2471/BLT.23.290975 PMC11362699

[40] Adair JG. The Hawthorne effect: A reconsideration of the methodological artifact. J Appl Psychol. 1984 May;69(2):334–45.

[41] Institut National de la Statistique (INSTAT), Cellule de Planification et de Statistique du Secteur Santé, Développement Social et Promotion de la Famille (CPS/SS-DS-PF), et ICF. Septième Enquête Démographique et de Santé au Mali 2023–2024. Indicateurs Clés. [Internet]. Bamako, Mali, et Rockville, Maryland, USA: INSTAT, CPS/SS-DS-PF, et ICF; 2024. Available from: https://dhsprogram.com/publications/publication-PR158-Preliminary-Reports-Key-Indicators-Reports.cfm

[42] Oron AP, Burstein R, Mercer LD, Arzika AM, Kalua K, Mrango Z, Effect Modification by Baseline Mortality in the MORDOR Azithromycin Trial. Am J Trop Med Hyg. 2020 Sep;103(3):1295–300. 30734696 10.4269/ajtmh.18-1004 PMC7470539

[43] Rolfe RJ, Shaikh H, Tillekeratne LG. Mass drug administration of antibacterials: weighing the evidence regarding benefits and risks. Infect Dis Poverty. 2022 Jun 30;11(1):77. 35773722 10.1186/s40249-022-00998-6 PMC9243730

[44] REACH Mali begins national azithromycin distribution in Ségou – REACH [Internet]. 2024 [cited 2025 Feb 24]. Available from: https://reachnetwork.africa/en/reach-mali-launch/

[45] Azithromycin for child survival in Niger – implementation and research – REACH [Internet]. 2024 [cited 2025 Feb 24]. Available from: https://reachnetwork.africa/en/azithromycin-for-child-survival-in-niger-implementation-and-research/

[46] SARMAAN II planning to treat 18 million children in 11 states in Nigeria – REACH [Internet]. 2025 [cited 2025 May 27]. Available from: https://reachnetwork.africa/en/sarmaan-18-million-children/

