# Supplementary material for: Mass Azithromycin Provision for Infants in Mali and Survival
Source: N Engl J Med. Author manuscript; Available in PMC 2026 Sep 2. (PMC13537731; doi:10.1056/NEJMoa2504644)
Supplement: Supplementary file 1 [file NIHMS2094101-supplement-supplement.pdf]

Contents

Supplementary Appendix 1: Details on community involvement in the randomization process 3

Figure S1: Trial flow chart – Extended version 4

Figure S2. Map of Trial villages by Study Group 5

Figure S3. Mortality difference between the control and biannual azithromycin groups, among predefined sub-groups of infants, who were 1 – 11-months old at the time of mass-drug administration, according to individual and household characteristics 6

Figure S4. Mortality difference between predefined sub-groups in the control and quarterly azithromycin groups, among infants, who were 1 – 11-months old at the time of mass-drug administration, according to ecological characteristics 7

Figure S5. Mortality difference between predefined sub-groups in the control and biannual azithromycin groups, among infants, who were 1 – 11-months old at the time of mass-drug administration, according to ecological characteristics 8

Table S1: Effect modifier categorization 9

Table S2. Success of infant treatment with study drug, by trial arm 10

Table S3. Number of recorded infant deaths, by round of mass drug administration (MDA) and study group 11

Table S4. Between-cluster standard deviation of mortality rates and coefficient of variation by arm and randomization strata 12

Table S5. All-cause mortality among participants who were 12-59 months old at the time of mass-drug administration 13

Table S6. Representativeness and Relevance of Study Participants in the Context of Infant and Child Mortality in Mali and the Sahel. 14

References 15

Supplementary Appendix 1: Details on community involvement in the randomization process

The public allocation events for randomization involved gathering village leaders and district officials at centrally organized meetings within the study districts. The principal investigator from the participating Malian research institution, along with the coordinating team, welcomed participants, reiterated the study objectives, and addressed questions from the audience.

The allocation procedure—drawing lottery tickets to assign villages to study groups—was then explained in detail. The importance of masking was emphasized, including the fact that the meaning of each letter code (i.e., treatment assignment) would remain unknown until the trial's conclusion.

All explanations were provided in French as well as in the local Bambara language to ensure full understanding. During the event, village chiefs personally drew the lottery tickets, and the letter code drawn was announced aloud for full transparency. Further details are available in the trial's Standard Operating Procedures at lakana.org.

Figure S1: Trial flow chart – Extended version

The term "compounds" refers to local residential groupings of multiple households in the study setting.

Abbreviations: MDA mass drug administration; PYR person-years at risk.

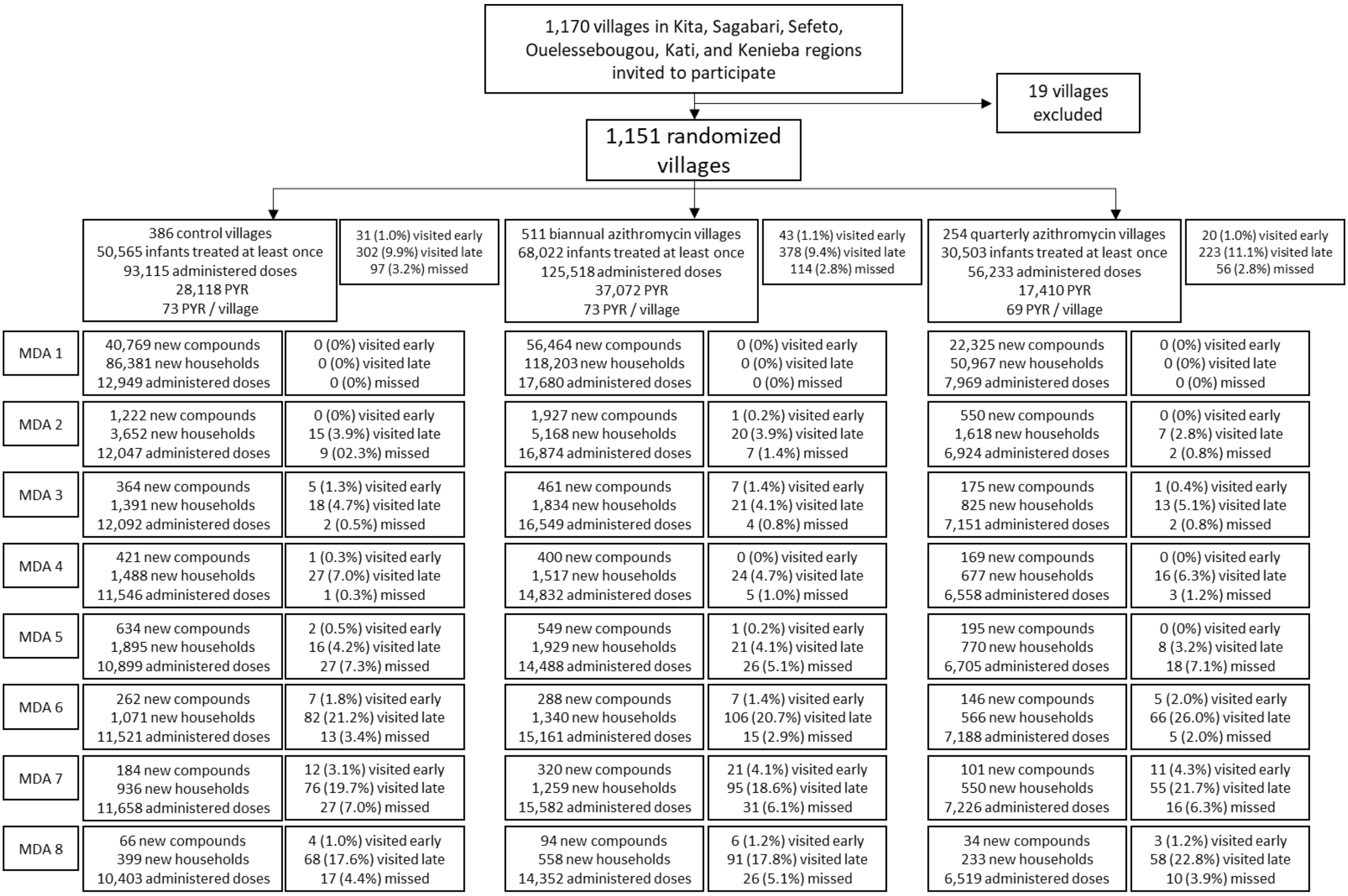

Figure S2. Map of Trial villages by Study Group

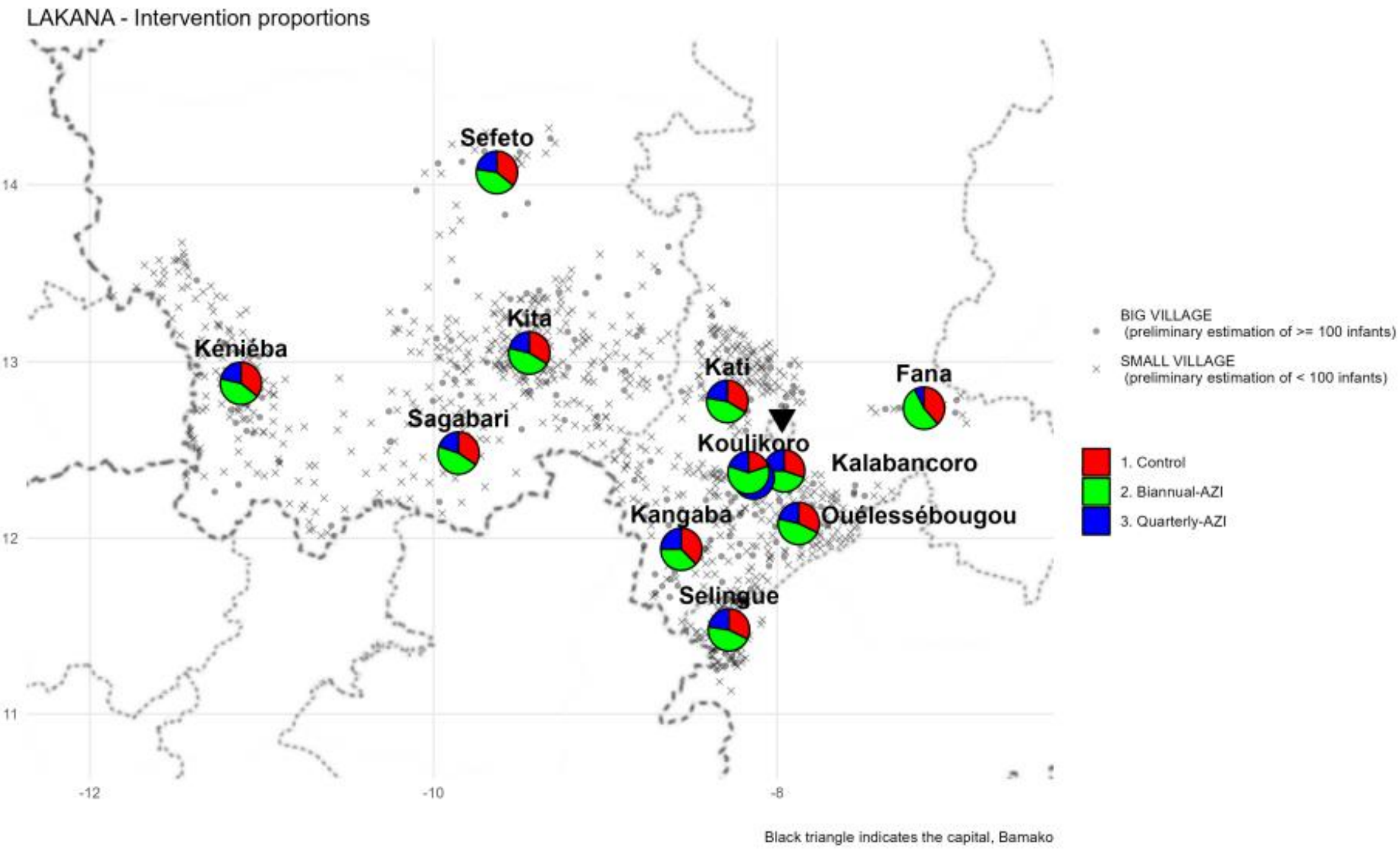

Figure S3. Mortality difference between the control and biannual azithromycin groups, among predefined sub-groups of infants, who were 1 – 11-months old at the time of mass-drug administration, according to individual and household characteristics

Abbreviations: AZI azithromycin; CI confidence interval; MDA mass drug administration; PYR person-years at risk; SMC Seasonal Malaria Chemoprevention; WASH Water, Sanitation, and Hygiene; WAZ Weight-for-Age Z-score.

Variable	BIANNUAL-AZI Deaths (PYR)	CONTROL Deaths (PYR)	Incidence rate ratio (95% CI)
Age at the time of MDA, mo			
1-5	130 (13,888.6)	128 (10,555.7)	0.77 (0.59, 1.00)
6-11	296 (22,155.5)	199 (16,728.4)	1.09 (0.88, 1.34)
Sex			
Male	237 (18,912.0)	159 (14,411.7)	1.14 (0.91, 1.43)
Female	200 (18,160.1)	176 (13,706.2)	0.87 (0.69, 1.09)
WAZ			
>= -2	123 (14,170.2)	88 (10,506.7)	0.99 (0.65, 1.50)
< -2	56 (4,025.8)	42 (2,979.2)	1.01 (0.75, 1.36)
SMC			
Given 3 months before	10 (837.9)	2 (412.2)	2.80 (0.61, 12.91)
Not given 3 months before	427 (36,374.9)	333 (27,825.4)	0.94 (0.79, 1.13)
Household asset index			
Above median	258 (24,413.7)	203 (18,759.1)	0.98 (0.79, 1.22)
Below median	179 (12,658.4)	132 (9,358.8)	1.01 (0.79, 1.30)
wash index			
Above median	292 (24,223.7)	235 (18,049.2)	0.93 (0.76, 1.14)
Below median	145 (12,848.4)	100 (10,068.7)	1.14 (0.86, 1.51)

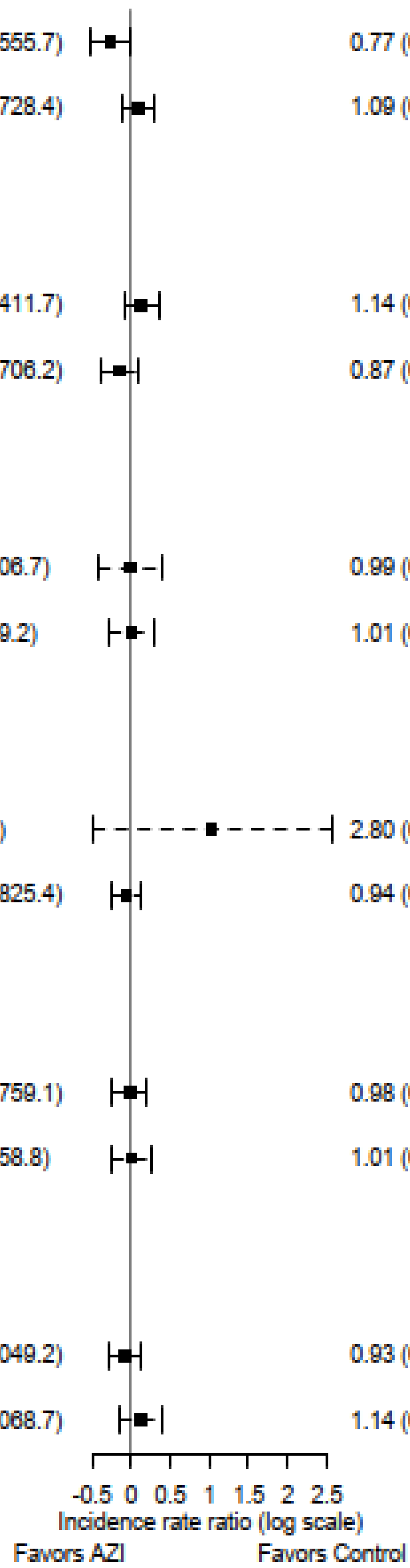

Figure S4. Mortality difference between predefined sub-groups in the control and quarterly azithromycin groups, among infants, who were 1 – 11-months old at the time of mass-drug administration, according to ecological characteristics

Abbreviations: AZI azithromycin; CI confidence interval; MDA mass drug administration; PYR person-years at risk.

Variable	QUARTERLY-AZI Deaths (PYR)	CONTROL Deaths (PYR)	Incidence rate ratio (95% CI)
Seasonality			
Rainy Season	63 (5,320.9)	121 (9,112.0)	0.89 (0.64, 1.24)
Non-rainy Season	115 (10,665.9)	174 (16,831.3)	1.04 (0.80, 1.36)
District of residence			
Kita	83 (5,716.4)	136 (7,453.2)	0.79 (0.57, 1.09)
Sagabari	5 (720.0)	20 (895.6)	0.30 (0.10, 0.89)
Sefeto	2 (550.9)	14 (1,086.3)	0.27 (0.06, 1.28)
Koulikoro	3 (386.4)	3 (184.3)	0.51 (0.08, 3.36)
Kalabancoro	2 (271.6)	8 (721.9)	0.58 (0.11, 3.08)
Kati	35 (2,768.2)	55 (5,964.5)	1.34 (0.84, 2.16)
Ouelessebougou	25 (1,743.8)	24 (3,754.7)	2.26 (1.22, 4.20)
Kenieba	25 (1,863.5)	25 (3,185.6)	1.58 (0.84, 2.97)
Fana	0 (27.8)	6 (219.5)	
Kangaba	12 (2,651.5)	27 (3,213.0)	0.55 (0.26, 1.19)
Selingue	4 (709.4)	17 (1,439.2)	0.49 (0.16, 1.51)
Order of MDA in a village			
1st	32 (2,622.5)	49 (4,141.4)	1.08 (0.67, 1.74)
2nd	27 (2,468.9)	59 (4,224.4)	0.76 (0.46, 1.24)
3rd	24 (2,323.8)	36 (3,657.9)	1.13 (0.64, 1.97)
4th	21 (1,995.6)	41 (3,448.5)	1.04 (0.58, 1.86)
5th	27 (2,212.6)	43 (3,581.6)	1.09 (0.64, 1.85)
6th	34 (2,095.0)	44 (3,295.3)	1.23 (0.75, 2.02)
7th	20 (1,983.1)	41 (3,042.0)	0.69 (0.38, 1.27)
8th	11 (1,786.8)	22 (2,846.3)	0.81 (0.37, 1.77)
Distance to Nearest Health Facility			
< 5km	101 (8,240.4)	172 (12,372.6)	0.96 (0.69, 1.34)
>= 5km	81 (8,391.8)	142 (14,778.5)	0.90 (0.68, 1.19)
National outreach strategy			
Standard	111 (8,425.8)	168 (12,417.2)	0.85 (0.60, 1.20)
Advanced	81 (8,566.9)	163 (15,462.3)	0.99 (0.75, 1.30)

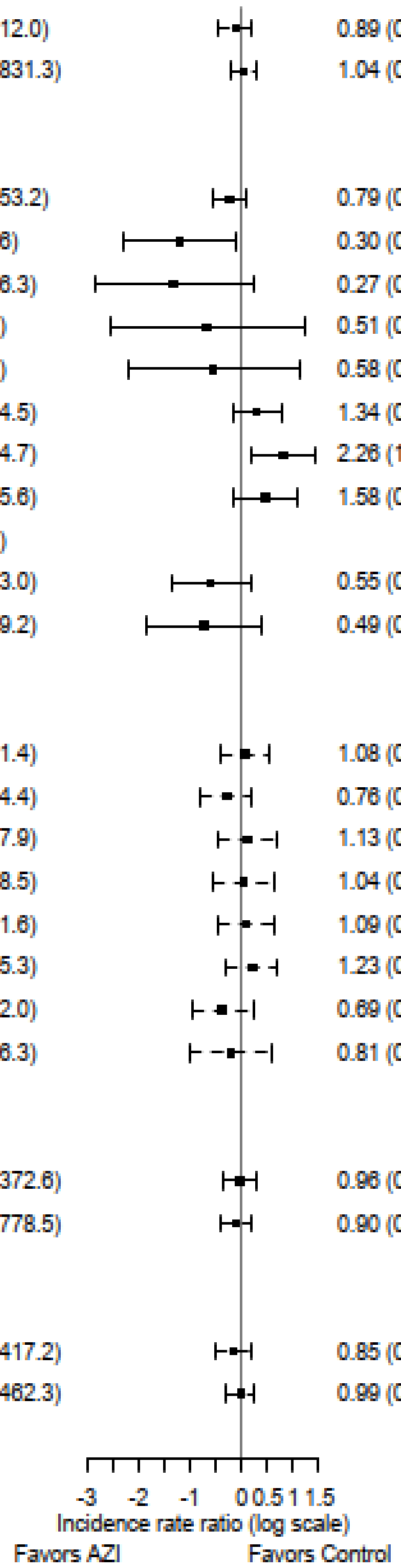

Figure S5. Mortality difference between predefined sub-groups in the control and biannual azithromycin groups, among infants, who were 1 – 11-months old at the time of mass-drug administration, according to ecological characteristics

Abbreviations: AZI azithromycin; CI confidence interval; MDA mass drug administration; PYR person-years at risk.

Variable	BIANNUAL-AZI Deaths (PYR)	CONTROL Deaths (PYR)	Incidence rate ratio (95% CI)
**Seasonality**			
Rainy Season	153 (11,573.2)	121 (9,112.0)	1.01 (0.77, 1.31)
Non-rainy Season	216 (22,620.7)	174 (16,831.3)	0.93 (0.74, 1.17)
**District of residence**			
Kita	195 (10,352.8)	136 (7,453.2)	1.03 (0.80, 1.33)
Sagabari	12 (879.5)	20 (895.6)	0.61 (0.27, 1.38)
Sefeto	3 (1,176.4)	14 (1,086.3)	0.19 (0.05, 0.70)
Koulikoro	10 (659.7)	3 (184.3)	0.98 (0.23, 4.26)
Kalabancoro	6 (1,482.2)	8 (721.9)	0.35 (0.10, 1.26)
Kati	81 (6,367.5)	55 (5,964.5)	1.43 (0.96, 2.14)
Ouelessebougou	30 (4,002.8)	24 (3,754.7)	1.13 (0.63, 2.04)
Kenieba	28 (3,526.9)	25 (3,185.6)	0.94 (0.51, 1.72)
Fana	12 (2,011.9)	6 (219.5)	0.19 (0.06, 0.63)
Kangaba	34 (3,823.9)	27 (3,213.0)	1.12 (0.62, 2.01)
Selingue	26 (2,788.5)	17 (1,439.2)	0.82 (0.42, 1.58)
**Order of MDA in a village**			
1st	49 (5,570.7)	49 (4,141.4)	0.75 (0.49, 1.14)
2nd	72 (5,651.7)	59 (4,224.4)	0.82 (0.56, 1.20)
3rd	44 (5,048.4)	36 (3,657.9)	0.87 (0.54, 1.42)
4th	57 (4,400.3)	41 (3,448.5)	0.95 (0.59, 1.53)
5th	68 (4,539.6)	43 (3,581.6)	1.18 (0.76, 1.83)
6th	62 (4,274.1)	44 (3,295.3)	1.09 (0.70, 1.68)
7th	50 (4,129.5)	41 (3,042.0)	0.97 (0.61, 1.56)
8th	35 (3,598.5)	22 (2,846.3)	1.34 (0.75, 2.39)
**Distance to Nearest Health Facility**			
< 5km	257 (17,227.5)	172 (12,372.6)	0.87 (0.65, 1.15)
>= 5km	156 (18,379.5)	142 (14,778.5)	1.10 (0.88, 1.36)
**National outreach strategy**			
Standard	252 (16,366.8)	168 (12,417.2)	0.80 (0.61, 1.07)
Advanced	185 (20,618.7)	163 (15,462.3)	1.15 (0.92, 1.44)

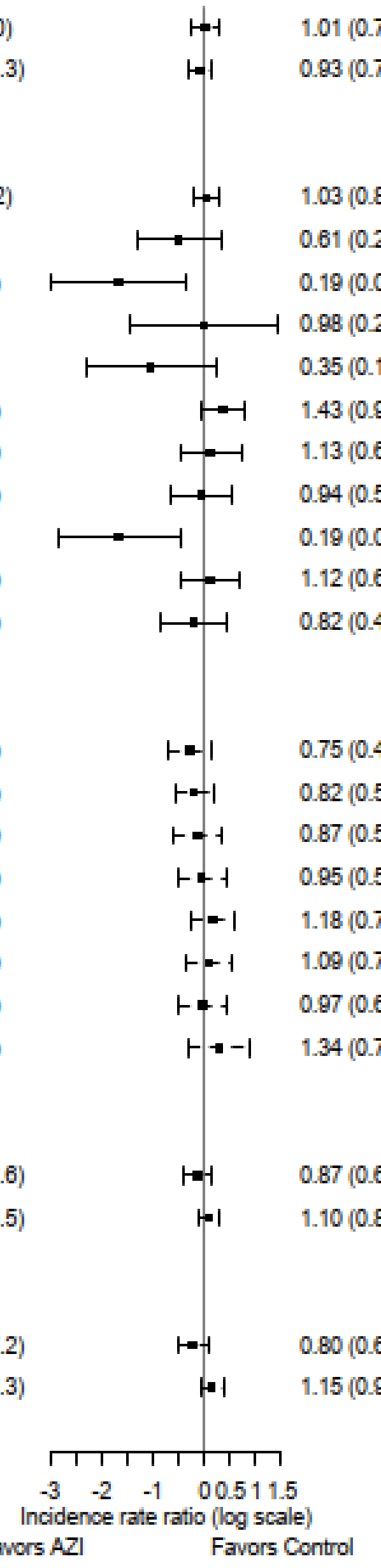

Table S1: Effect modifier categorization

**Effect modifier**	**Categorization**
Child’s age at MDA	1-5 months<br>6-11 months
Sex	Male<br>Female
Weight-for-age Z-score (WAZ)	< -2<br>>= -2
Season	Rainy<br>Non-Rainy
Seasonal Malaria Chemoprevention (SMC) given to the child within 3 months of an MDA	Given<br>Not Given
Order of MDA	1-8
District of residence	Kita<br>Kati<br>Fana<br>Kalabancoro<br>Sefeto<br>Sagabari<br>Ouéléssébougou<br>Kéniéba<br>Selingue<br>Koulikoro<br>Kangaba
Distance from the nearest health facility in kilometers	< 5km<br>>= 5km
Household Asset index	Below median<br>Above median
Water, sanitation, and hygiene (WASH) index	Below median<br>Above median
National outreach strategy,<br>categorizing villages based on the effort needed to deliver health services, determined by their distance from the nearest health facility	Standard<br>Advanced

Table S2. Success of infant treatment with study drug, by trial arm

	Control	Biannual azithromycin	Quarterly azithromycin
Number of infants treated at least once	50,565	68,022	30,503
Total number of recorded treatment events	93,115	125,518	56,233
Number (%) of missed treatments			
Temporarily absent	2,894 (3.0%)	3,664 (2.8%)	2,038 (3.5%)
Weighing less than 3 kg[1]	161 (0.3%)	229 (0.2%)	95 (0.3%)
Too ill and referred	109 (0.1%)	104 (0.1%)	54 (0.1%)
Considered allergic	77 (0.2%)	87 (0.1%)	62 (0.2%)
Parental refusal	201 (0.2%)	216 (0.2%)	103 (0.2%)
Number (%) of incorrect treatments			
Incorrect letter code	155 (0.2%)	66 (0.1%)	52 (0.1%)
Incorrect preparation (placebo vs azithromycin)	0 (0%)	41 (0.03%)	0 (0%)
Number (%) of second dose due to vomiting	740 (0.8%)	753 (0.6%)	437 (0.8%)

[1]The minimum thresholds of 1 month of age and 3 kg in weight were implemented as safety measures to mitigate the risk of adverse events, including infantile hypertrophic pyloric stenosis (IHPS), a rare but serious condition associated with macrolide use in neonates.

Table S3. Number of recorded infant deaths, by round of mass drug administration (MDA) and study group

	All	Control	Biannual azithromycin	Quarterly azithromycin
MDA 2	130	49	49	32
MDA 3	158	59	72	27
MDA 4	104	36	44	24
MDA 5	119	41	57	21
MDA 6	138	43	68	27
MDA 7	140	44	62	34
MDA 8	111	41	50	20
Visit 9	68	22	35	11
Total	968	335	437	196

Table S4. Between-cluster standard deviation of mortality rates and coefficient of variation by arm and randomization strata

	Deaths	Person-years-at-risk	Mean Mortality rate (mean of all villages' deaths/person-years-at-risk)	Standard deviation of village mortality rates	Coefficient of variation[1]
Overall	968	82599.4	0.0133	0.0244	1.83
Control, all villages	335	28117.9	0.0127	0.0219	1.72
Small village strata[2]	191	15823.5	0.0126	0.0232	1.85
Large village strata[3]	144	12294.4	0.0133	0.0145	1.09
Biannual azithromycin, all villages	437	37072.1	0.0140	0.0270	1.92
Small village strata	278	20778.2	0.0149	0.0291	1.96
Large village strata	159	16293.9	0.010	0.0114	1.14
Quarterly azithromycin, all villages	196	17409.5	0.0127	0.0227	1.78
Small village strata	119	9434.9	0.0131	0.0243	1.85
Large village strata	77	7974.6	0.011	0.0124	1.16

[1]Coefficient of variation calculated by dividing the standard deviation of village mortality rates with the mean mortality rate.

[2]Below 100 infants, based on national population estimates.

[3]Above 100 infants, based on national population estimates.

Table S5. All-cause mortality among participants who were 12-59 months old at the time of mass-drug administration

	Control	Biannual azithromycin	Quarterly azithromycin	Quarterly vs Biannual
Number of deaths	312	452	203	
Number of PYR	68,566.4	93,224.4	42,931.6	
Number of deaths/1000 PYR	4.6	4.9	4.7	
Incidence rate ratio[1]	REF	1.02	0.97	0.95
(95% CI)		(0.84 to 1.24)	(0.77 to 1.22)	(0.76 to 1.18)
p-value		0.60	0.40	0.31
Incidence rate difference	REF	0.13	-0.16	-0.29
(95% CI)		(-0.86 to 1.11)	(-1.32 to 1.00)	(-1.38 to 0.82)
p-value		0.80	0.79	0.61

[1]Adjusted for stratified randomization factor (village size category).

Abbreviations: CI confidence interval; REF reference; PYR person-years at risk.

Table S6. Representativeness and Relevance of Study Participants in the Context of Infant and Child Mortality in Mali and the Sahel.

**Category**	**Detail**
Condition under investigation	Infant and child mortality
Special considerations related to	
Age	Children under five face a high risk of mortality, particularly newborns and infants(1).<br>In Mali and across the Sahel region, leading causes of infant mortality are infectious diseases. According to UNICEF, Mali's infant mortality rate was approximately 62 deaths per 1,000 live births in 2020(2).
Sex and gender	Globally, boys under five have a higher mortality risk than girls.<br>Mortality is higher in Mali in male compared to female infants (67 deaths per 1000 live births in male infants versus 57 deaths per 1000 live births in female infants in 2020 per UNICEF)(2)
Geography	Sub-Saharan Africa includes all 20 countries with the highest under-five mortality rates globally(3).
Other considerations	Within sub-Saharan Africa, the Sahel region has among the highest rates of infant and child mortality.
Overall representativeness of this trial	The trial participants reflect the expected age and sex distributions for this population. The infant's biological sex (male or female) was reported by the caregiver. All participants were enrolled in Mali, and the study was conducted in rural communities, aligning with national demographics—nearly 75% of Mali's population resides in rural areas(4). The study population also reflects key risk factors—such as limited access to healthcare—that are common in rural Mali and throughout the Sahel, supporting the representativeness of the sample to the broader high-risk child population in the region.<br><br>A population-based census was used to map the study area and served as the basis for household enumeration and participant enrollment. The study team visited all households in the area, with minimal refusals or loss to follow-up, ensuring the study population was representative of the regional child population.<br><br>The study was conducted in central and western regions of Mali, which are less affected by armed conflict than northern Mali. As such, mortality rates in the study areas are likely lower than those in conflict-affected northern populations. Nonetheless, recent national data and findings from other trials indicate a general decline in child mortality rates in Mali and neighboring Sahelian countries(4–8)—a trend observed in our study.

References

1. Sharrow D, Hug L, You D, Alkema L, Black R, Cousens S, et al. Global, regional, and national trends in under-5 mortality between 1990 and 2019 with scenario-based projections until 2030: a systematic analysis by the UN Inter-agency Group for Child Mortality Estimation. Lancet Glob Health. 2022 Feb;10(2):e195–206.

2. UNICEF. Mali – UNICEF DATA [Internet]. Available from: https://data.unicef.org/country/mli/

3. United Nations Inter-agency Group for Child Mortality Estimation (UN, IGME). Levels & Trends in Child Mortality: Report 2024 – Estimates developed by the United Nations Inter-agency Group for Child Mortality Estimation. New York: United Nations Children's Fund; 2024.

4. Institut National de la Statistique (INSTAT), Cellule de Planification et de Statistique du Secteur Santé, Développement Social et Promotion de la Famille (CPS/SS-DS-PF), et ICF. Septième Enquête Démographique et de Santé au Mali 2023–2024. Indicateurs Clés. [Internet]. Bamako, Mali, et Rockville, Maryland, USA: INSTAT, CPS/SS-DS-PF, et ICF; 2024. Available from: https://dhsprogram.com/publications/publication-PR158-Preliminary-Reports-Key-Indicators-Reports.cfm

5. Oldenburg CE, Ouattara M, Bountogo M, Boudo V, Ouedraogo T, Compaoré G, et al. Mass Azithromycin Distribution to Prevent Child Mortality in Burkina Faso: The CHAT Randomized Clinical Trial. JAMA. 2024 Feb 13;331(6):482–90.

6. O'Brien KS, Arzika AM, Amza A, Maliki R, Aichatou B, Bello IM, et al. Azithromycin to Reduce Mortality — An Adaptive Cluster-Randomized Trial. N Engl J Med. 2024 Aug 22;391(8):699–709.

7. Liu J, Treleaven E, Whidden C, Doumbia S, Kone N, Beydi Cisse A, et al. Home visits versus fixed-site care by community health workers and child survival: a cluster-randomized trial, Mali. Bull World Health Organ. 2024 Sep 1;102(9):639–49.

8. Sié A, Ouattara M, Bountogo M, Boudo V, Ouedraogo T, Compaoré G, et al. Azithromycin during Routine Well-Infant Visits to Prevent Death. N Engl J Med. 2024 Jan 17;390(3):221–9.
